# Authors response to correspondence regarding paper entitled Syphilitic hepatitis: a case report and review of the literature

**DOI:** 10.1186/s12876-020-01497-4

**Published:** 2020-11-17

**Authors:** Jiaofeng Huang, Bo Wan, Mingfang Wang, Yueyong Zhu, Su Lin

**Affiliations:** 1 Department of Hepatology, Hepatology Research Institute, The First Affiliated Hospital, Fujian Medical University, No. 20, Chazhong Road, Taijiang District, Fuzhou, 350001 Fujian China; 2 Faculty of Life Sciences and Medicine, King’s College London, London, SE1 1UL UK; 3 Guy’s and St Thomas’ NHS Foundation Trust, London, UK

**Keywords:** Syphilis, Hepatitis, Rashes

## Abstract

In the correspondence from Abdurrahman et al., they raised three main concerns and critiques of our recently published article entitled “Syphilitic hepatitis: a case report and review of the literature”. First question pertains to the timing of dermatology opinion, second regarding the history of sexual exposure, and lastly regarding the treatment duration of syphilitic hepatitis. We thank the authors for their constructive comments and would like to answer these questions in detail.

We appreciate the detailed review and commentary on our manuscript [1] from Abdurrahman et al. [2]. The first concern is regarding the timing of dermatology consult and syphilitic rashes. The patient described in our manuscript had two dermatology consultation. The first consult was at the local hospital when the rashes were noticed, and the second consult at our referral hospital upon admission. At both consultation with dermatologist, the opinion was that the rashes were secondary to an allergic reaction. The complete clinical examination of the genitals, palms and soles but did not reveal any skin lesions. Syphilitic rashes are known to occur in around 80% of patients with syphilitic hepatitis [3], and often present as multiple non-pruritic, erythematous, nonconfluent maculopapular lesions generally concentrated in the trunk, palms and soles of the feet. We agree with the authors that if the correct diagnosis of syphilitic hepatitis could have been made earlier, the invasive liver biopsy could have been avoided. This is precisely the reason why we decided to report this particular case.

Second, Abdurrahman et al. noted that we did not describe the patient's sexual history in detail, we agree with him on this point. In fact, the patient denied a history of unsafe sexual activity upon admission, which we duly stated in the manuscript. Interestingly, even after the syphilis infection was confirmed, patient still denied any history of unsafe sexual activity. However, even in the absence of a clear high risk sexual history, the antibody positivity was still powerful evidence of the diagnosis of Syphilis infection.

The third question Abdurrahman et al. raised is about the duration of antibiotics administered to patient for syphilitic hepatitis. The recommended duration of antibiotic administration is 2–4 weeks [4]. The reported patient was discharged after the first dose of penicillin injection from our hospital. At the second follow-up visit, patient informed us that he has received a 2-month penicillin injection at a local clinic. We too concure with Abdurrahman et al. that the treatment duration of 2 months was too long and is problematic at multiple levels, however we recorded and reported the case-report as matter of fact. Prolonged duration of treatment however would not change the diagnosis of syphilitic hepatitis.

In conclusion, although there are some deficits and missing information in our case report, the diagnosis of syphilis is quite clear. This case highlights an atypical presentation of syphilitic hepatitis and and we hope that readers would get better insight into this rare disease.

## Data Availability

Not applicable.
